# Comparison Study on the Adsorption Behavior of Chemically Functionalized Graphene Oxide and Graphene Oxide on Cement

**DOI:** 10.3390/ma13153274

**Published:** 2020-07-23

**Authors:** Min Wang, Hao Yao

**Affiliations:** 1 Key Laboratory of Building Safety and Energy Efficiency of the Ministry of Education, College of Civil Engineering, Hunan University, Changsha 410082, China; 2 School of Civil Engineering, Central South University, Changsha 410075, China

**Keywords:** adsorption behavior, chemically functionalized graphene oxide, graphene oxide, cement, flowability

## Abstract

Chemical functionalization of graphene oxide (GO) is one kind of advanced strategy to eliminate the negative effects on the flowability of cement with GO. The adsorption behavior of admixture on cement plays a vital role in the flowability of cement-based materials. Herein, the comparison study on the adsorption behavior (including adsorption amount, adsorption kinetics, adsorption isotherms and adsorption layer thickness) of three kinds of chemically functionalized graphene oxides (CFGOs) with different polyether amine branched-chain lengths and GO on cement is reported. The results of CFGOs and GO adsorption data on cement particles were all best fitted with the pseudo-second-order kinetic model, and also conformed to the Freundlich isothermal model, indicating that the adsorption of CFGOs and GO on cement both were multilayer type and took place in a heterogeneous manner. The adsorption of CFGOs and GO on cement was not just physical adsorption, but also engaged chemical adsorption. In contrast to GO, the adsorption behavior of CFGOs on cement represented a lesser adsorption amount, weaker adsorption capacity and thinner adsorption layer thickness. Moreover, the longer the branched-chain length of CFGOs, the greater the decreasing degrees of adsorption amount, adsorption capacity and adsorption layer thickness. Due to the consumption of the carboxyl group (-COOH) by chemical functionalization, the anchoring effect of CFGOs was weaker than GO, and the steric hindrance effect generated from branched-chains which weakened the van der Waals forces among CFGOs layers. Moreover, the steric hindrance effect strengthened with the increasing branched-chain length, thus preventing the cement particles from aggregation, which resulted in satisfactory flowability of CFGOs with incorporation of cement rather than GO.

## 1. Introduction

In recent years, carbon materials have been widely utilized to improve various properties of cement-based materials [1,2,3,4,5]. As a new kind of carbon material, graphene oxide (GO) is the intermediate of graphene, which has attracted much research attention because of its high reactivity, high specific surface area and excellent mechanical properties [6,7,8]. The existing research on the performance of cement-based materials considered the introduction of GO and exhibited the enormous potential to enhance the mechanical properties and durability of hardened cement-based materials [9,10,11,12,13,14,15,16,17,18,19,20]. Devi et al. [21] explored the compressive and tensile strengths of the mixtures with 0.08% GO, showing a better result compared to the rest of the mixes, and the sorptivity and permeability of the concrete with GO reduced with an increasing GO content. Huang et al. [22] reported that GO can considerably improve the compressive strength, flexural strength, and elasticity modulus of concrete, but GO can increase the shrinkage strain of concrete. However, most of these works were focused on the optimization of the dosage-dependence of GO on the microstructure and macro-performance on hardened cement-based materials without considering that GO has an adverse impact on the flowability of fresh cement [23,24,25,26]. The high percentage of GO demanded the higher percentage of polycarboxylate superplasticizer to balance the self-compacting nature. However, the adverse effects of polycarboxylate superplasticizer over dosage in the form of bleeding and segregation were observed during fresh properties [27]. There is no doubt that good flowability is the precondition of concrete agitation and pump transport, which is a key performance for cement-based materials in engineering applications.

In this context, how to overcome the flowability drawback of cement-based materials with incorporation of GO is of great significance. In our previous study, chemically functionalized graphene oxide (CFGOs) were synthesized through chemical reaction between GO and polyether amine with different molecular weights [28]. CFGOs increased the flowability of cement, which is in contrast to GO, and the flowability improved with the increase of CFGO dosages and polyether amine molecular weight. It is well known that adsorption of admixture on cement is a key step in large numbers of dispersion processes such as steric and electrical stabilization, which can be expected to be responsible for the dispersibility of admixture on the cement system. Hence, clearly understanding the adsorption behavior of admixture on cement is helpful to clarify the mechanisms behind the improvements and future formulations. For the negative effect of GO on the flowability of cement, the adverse impact on flowability of cement is induced by GO absorbed on cement surface, the van der Waals force among the GO layers led to the aggregation of cement particles [26]. However, the adsorption behavior of CFGOs on cement is still absent, the lack of comparative mechanism research on GO and CFGOs with cement limits the further application of GO and its derivatives. It is therefore reasonable to infer that the diametrically opposite effect of CFGOs and GO on the flowability of cement can directly be reflected in the different adsorption behavior of CFGOs and GO on cement. Therefore, it is necessary to research and compare the adsorption behavior of CFGOs and GO on cement to explore the essence of the CFGOs and GO with the incorporation of cement.

Herein, this paper aimed at understanding the comparison study on workability, mechanical properties and adsorption behavior of CFGOs and GO on cement. More specifically, the adsorption amount, adsorption isotherms, adsorption kinetics and adsorption layer thickness of CFGOs and GO were investigated with different concentrations, and the reasons for the opposite effect of CFGOs and GO on flowability were discussed. The results of this research demonstrate that the adsorption of CFGOs and GO on the surface of cement both were multilayer adsorption and took place in a heterogeneous manner. The adsorption of CFGOs and GO on cement was not just physical adsorption, but also engaged chemical adsorption. Compared with GO, CFGOs with incorporation of cement exhibited lesser adsorption amount, weaker adsorption capacity and thinner adsorption layer thickness. The information that provided by this work would not only reveal a clear mechanism of the different influence of CFGOs and GO on cement flowability, but also endow a valuable guidance for the application of GO and its derivatives on cement filed in the future.

## 2. Materials and Methods

### 2.1. Materials

Portland cement (Type 52.5R, Chinese National Standard GB/T 4131-2014) from Anhui Conch Cement Company Limited was used. The main chemical components are shown in Table 1.

**Figure 1:**
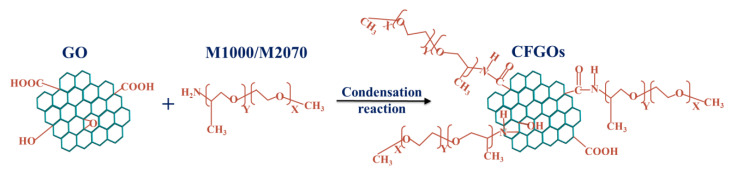
Schematic illustration of chemically functionalized graphene oxides (CFGOs).

Graphene oxide (GO) and chemically functionalized graphene oxides (CFGOs) are lab-made. The modified Hummer’s method was used to synthesize GO. CFGOs were prepared by the condensation reaction of -COOH/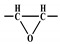 on GO and –NH_2_ in polyether amine. The chemical structure of GO and CFGOs was shown in Figure 1, and the detailly synthesized process of GO and CFGOs was described in our previous research [28].

The different types of CFGOs were obtained by grafting polyether amine (M1000/M2070) with different molecular weights onto GO. The molecular weights of M1000 and M2070 were 1000 and 2000, respectively. The monomer ratios of M1000 and M2070 for CFGO-1, CFGO-2 and CFGO-3 were 1:0, 1:1 and 0:1, respectively. The dosages of the GO and polyether amine in the different CFGOs are listed in Table 2.

**Figure 2:**
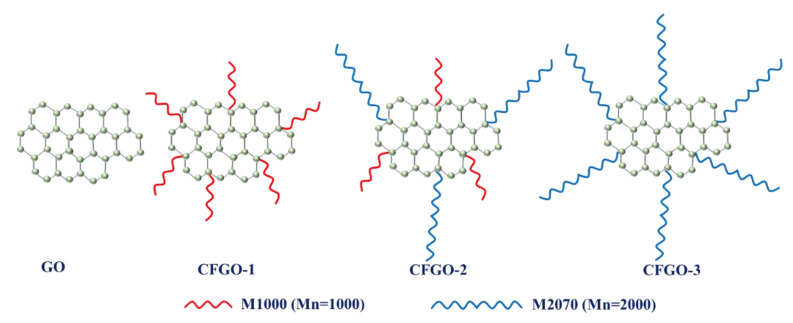
Schematic illustration of GO and CFGOs with different lengths of branched-chains.

As illustrated in our previous research [28], CFGOs were successfully synthesized by grafting polyether amine onto GO. In other words, GO and CFGOs represented the different chemical structure, CFGOs could be regarded as that of polyether amine branched on GO, as shown in Figure 1 and Figure 2. In the CFGO structure, polyether amine acted as the branched-chains, the length of branched-chains increased with the polyether amine molecular weight. Additionally, due to the dosages of reactants for CFGOs being different, the branched-chain length of CFGO-1 was shorter than that of CFGO-2 and CFGO-3, the branched-chain length of CFGO-3 was the longest.

### 2.2. Characterization

#### 2.2.1. Flowability Measurement of Cement Paste

The flowability of the cement paste with GO/CFGOs was measured according to the Chinese National Standard GB/T 8077–2000 [26,28]. At first, 300 g cement, 100 g water and GO/CFGOs were mixed with each other for 3 min. Then, the mixture was poured into a cone mold (base diameter of 60 mm, top diameter of 36 mm and height of 60 mm) in a cleaned and moist glass plate. Then, the mold is lifted at about 15 cm above the glass plate, and the fresh cement paste will collapse and spread. The parallel diameter of the spread was d_1_, and the vertical diameter was d_2_. The value of the (d_1_ + d_2_)/2 is the paste flowability. For time-dependent flowability testing, the cement paste was put back into the mold and covered with a wet towel after each measurement. In each test, the cement paste was stirred again for 2 min.

#### 2.2.2. Adsorption Experiment of GO/CFGOs on Cement

**Figure 3:**
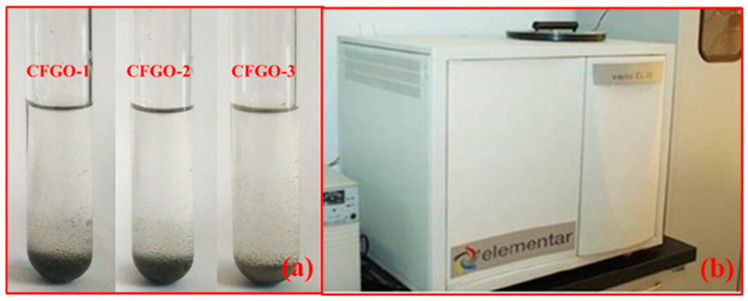
(**a**) The adsorption results of CFGOs on the surface of cement, (**b**) Apparatus for total-organic carbon test.

Standard aqueous solutions of GO/CFGOs with different concentrations (250, 300, 350, 400, 450, 500, 550, 600 and 650 mg/L) were prepared by dispersing GO/CFGOs in deionized water. The adsorption results of CFGOs on the surface of cement and apparatus for total-organic carbon test were shown in Figure 3, and the adsorption results of GO on the surface of cement were displayed in our previous research [26]. The screened cement powder (0.09 g) was added into beaker flask containing GO/CFGOs aqueous solutions (60 g). The stirring continued for different time (10, 20, 30, 60, 90 and 120 min) in the crystal oscillator (25 °C). Then, the mixture was vacuum filtered, and the GO/CFGOs supernatant concentration was measured by the total-organic carbon analyzer (TOC-II, Elementar Co., Frankfurt, German). The adsorption amount of GO/CFGO on the cement can be obtained by the following Equation (1):

(1) $$Q_{e}=\frac{{(C}_{o}-C_{t})V}{m}$$

where *Q_e_* (mg/g) presents the adsorption amount of the GO/CFGO by unit mass of cement; *C*_0_ (mg/L) and *C_t_* (mg/L) are the initial concentration and concentration of the GO/CFGO at *t* min; *V* (mL) represents the volume of the solution; *m* (g) represents the mass of cement.

#### 2.2.3. X-ray Photoelectron Spectroscopy (XPS) Measurement of GO/CFGOs on Cement

For the XPS measurement, the same concentrations (750 mg/L) of GO/CFGOs aqueous dispersion were prepared. Then, 0.09 g cement and 60 g GO/CFGOs aqueous dispersion was added into a beaker flask. For the pure cement sample, 0.09 g cement was added in 60 g water. The mixture was vibrated in the oven-controlled crystal oscillator at room temperature for 5 h. Finally, the mixture was vacuum filtered. The filter cake can be used to test the adsorption layer thickness of GO/CFGOs on the surface of cement. The XPS analysis was carried out on an AXIS–Ultra instrument from Kratos Analytical (Manchester, UK) using monochromatic Al Ka radiation (225 W, 15 mA, 15 kV) and low–energy electron flooding for charge compensation. To compensate for surface charges effects, binding energies were calibrated using C1s hydrocarbon peak at 284.80 eV.

#### 2.2.4. Mechanical Properties of Cement Paste with GO/CFGOs

Firstly, GO/CFGOs and water were added to a stainless-steel container in turn and mixed well. Secondly, the mixture of GO/CFGOs and water was divided into three equal parts. Finally, these three-part mixtures were added into cement in time intervals of 3 min and mixed well. Three specimens for each test were immediately poured into the mold of 40 mm × 40 mm × 160 mm size. The specimens were allowed to cure in the mold for 24 h. After 24 h, the specimens were cured in water at 20 ± 2 °C for 6 days and 21 days. The flexural strength was determined using a DKZ-500 concrete three-point flexural strength tester (Tianjin, China) at a load increasing rate of 0.05 KN/s. The compressive strength was tested using a JES-300 concrete compressive strength tester (Tianjin, China) with an increase rate of 2.4~2.6 MPa/s. To check for reproducibility of the results, three/six samples were tested each for flexural/compressive test, respectively and averaged the results.

## 3. Results and Discussion

### 3.1. Dispersibility of GO/CFGOs

In order to investigate the dispersibility of GO and CFGOs with different dosages in cement paste, a mini-slump test for the cement paste was implemented, and the water–cement ratio was 1:3. As listed in Table 3, we could observe that as an admixture, GO led to the decreasing flowability of cement paste, and the flowability of cement paste reduced with the dosages of GO. In the case where the GO dosage was 0.05%, the flowability of cement paste was 180.3 mm, and the reduction in flowability was 18.6%. However, for CFGOs, the flowability tendencies of cement paste were all contrary to GO, which increased with the dosages of CFGOs. This is direct evidence that the chemically functionalized process of GO was a very efficient way to change the GO adsorption behavior on the cement, and this is further elaborated in the following section. At the same CFGO dosage, the dispersibility of CFGO-3 was superior to CFGO-2 and CFGO-1. Moreover, CFGO-1 showed the worst dispersibility in cement paste. In the case where the CFGO dosages were 0.05%, the flowability of CFGO-3, CFGO-2 and CFGO-1 with the incorporation of cement paste was 288.5 mm, 253.5 mm and 245.2 mm, respectively. As a result, the flowability of CFGO-3, CFGO-2 and CFGO-1 with the incorporation of cement paste increased by 30.2%, 11.4% and 11.7%, respectively. In a word, these results indicated that CFGOs were in opposite to GO, CFGOs could increase the flowability of cement, and the CFGOs with longer branched-chains bring better flowability for cement.

**Figure 4:**
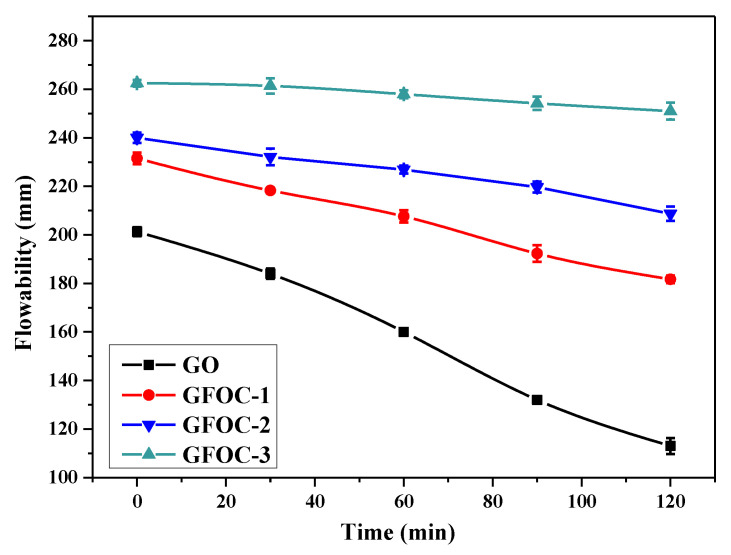
Flowability of cement paste with time at 0.03 wt.% dosage of GO, CFGO-1, CFGO-2 and CFGO-3.

The flowability retention behavior of cement paste with GO, CFGO-1, CFGO-2 and CFGO-3 was investigated per 30 min for 120 min at 0.03 wt.% dosage. As shown in Figure 4, the flowability of cement paste with GO rapidly decreased with time, the flowability reduced by 20.0% at 60 min and 44.4% at 120 min, respectively. For cement paste with CFGO-1, CFGO-2 and CFGO-3, all of them had good flowability retention ability. Furthermore, the flowability retention ability was strong with the increase of the branched-chain length. The flowability of cement paste with CFGO-3 fell only 1.7% after 60 min, and the same value of CFGO-1 was 10.3%.

### 3.2. Flexural Strength of Cement Paste with GO/CFGOs

**Figure 5:**
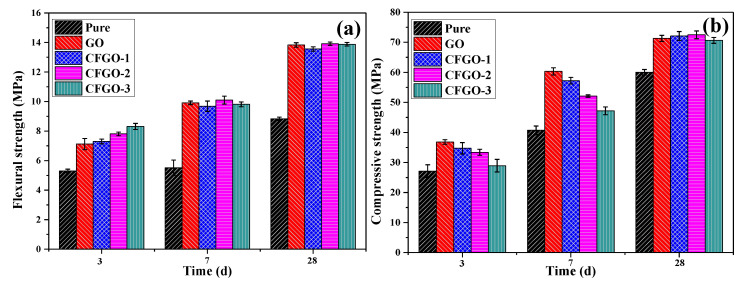
(**a**) Flexural strength and (**b**) compressive strength of cement paste with different curing time at 0.03 wt.% dosage.

The flexural strength and compressive strength of cement paste with different curing time at 0.03 wt.% GO and CFGO dosage were shown in Figure 5. The results indicated that the flexural strength and compressive strength of cement paste increased after the addition of GO and CFGOs. After 3d, the flexural strength of cement paste with GO was lower than CFGOs, and increased with branched-chain length (Figure 5a). At 7d and 28d, there was little change in flexural strength of cement paste with GO and CFGOs. This means that the chemically functionalized process also improved the toughening action of GO in the cement matrix. As shown in Figure 5b, the compressive strength of cement paste with GO was higher than CFGOs, and decreased with branched-chain length at 3d and 7d. The compressive strength of cement paste GO and CFGOs was almost equal at 28d.

### 3.3. Adsorption Amount of GO/CFGOs on Cement

**Figure 6:**
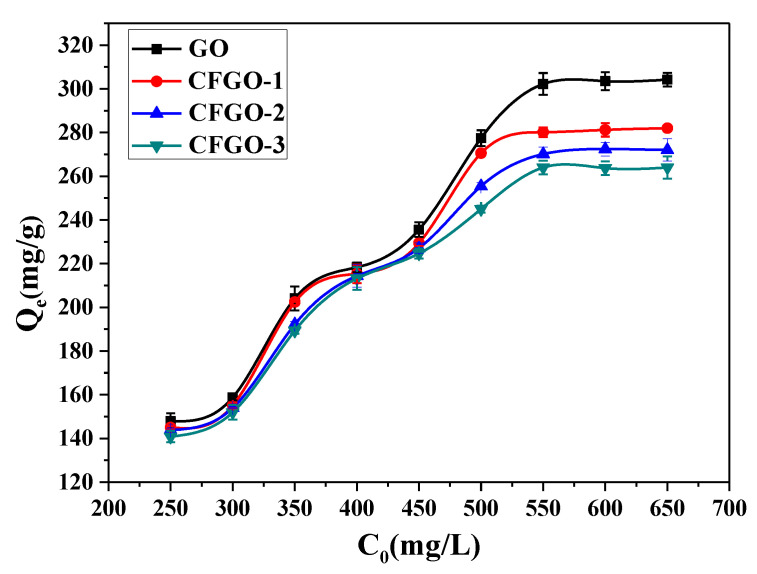
Increase in adsorption amount (*Q_e_*) with concentration (*C*_0_).

Figure 6 demonstrates the effect of the GO and CFGO concentration (*C*_0_) on *Q_e_* at adsorption equilibrium. As shown in Figure 6, it is observed that all of the *Q_e_* rapidly raised with the increase of *C*_0_, and the *Q_e_* of GO and CFGOs approached saturation when C_0_ was higher than 550 mg/L. In the case where the C_0_ was 500 mg/L, the corresponding *Q_e_* of GO was 277.44 mg/g; for CFGOs, the same values of CFGO-1, CFGO-2 and CFGO-3 were 270.56 mg/g, 255.45 mg/g and 245.00 mg/g, respectively. At the higher concentration, the *Q_e_* of GO stayed around 300.00 mg/g; for CFGOs, the *Q_e_* of CFGO-1, CFGO-2 and CFGO-3 were about 280.00 mg/g, 270.00 mg/g and 260.00 mg/g, respectively.

These results elucidate that the adsorption amount of GO on cement was higher than that of CFGOs, and the adsorption amount of CFGOs on cement decreased with the increasing length of branched-chains.

### 3.4. Adsorption Kinetics of GO/CFGOs on Cement

Pseudo-first-order and pseudo-second-order rate models are the most widely used models in solid–liquid interface adsorption [26,29,30,31], therefore, we applied both the pseudo-first-order and pseudo-second-order rate models to the adsorption data of GO/CFGOs to explore the time-dependent adsorption process. These two equations are listed as Equations (2) and (3):

(2) $$log\left(Q_{e}-Q_{t}\right)=logQ_{t}-\frac{K_{1}t}{2.303}$$

(3) $$\frac{t}{Q_{t}}=\frac{1}{{2K}_{2}{Q_{e}}^{2}}+\frac{t}{Q_{e}}$$

where *K*_1_ (min^−1^) presents the equilibrium rate constant of pseudo-first-order model; *K*_2_ (g·mg^−1^·min^−1^) presents the equilibrium rate constant of pseudo-first-order model; *Q_e_* (mg·g^−1^) is the adsorption amount of equilibrium adsorption; *Q_t_* (mg·g^−1^) represents the adsorption amount at time *t* (min).

**Figure 7:**
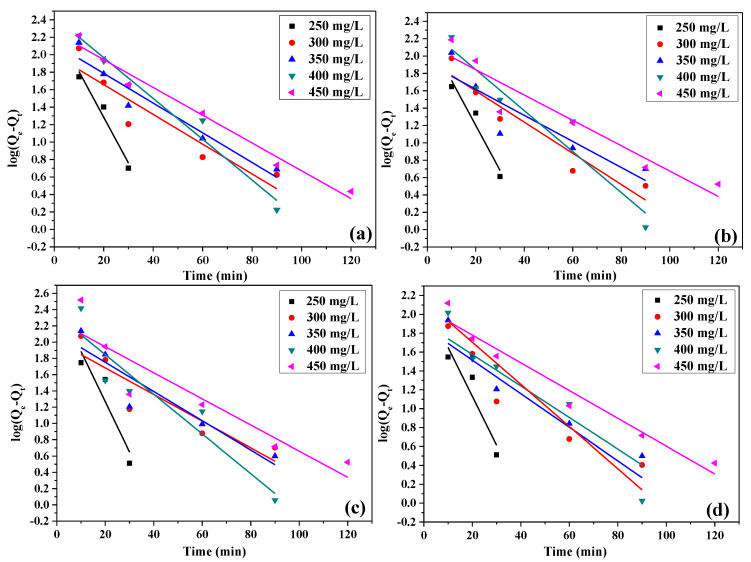
The pseudo-first-order kinetic model for (**a**) GO, (**b**) CFGO-1, (**c**) CFGO-2 and (**d**) CFGO-3 adsorption on cement.

**Figure 8:**
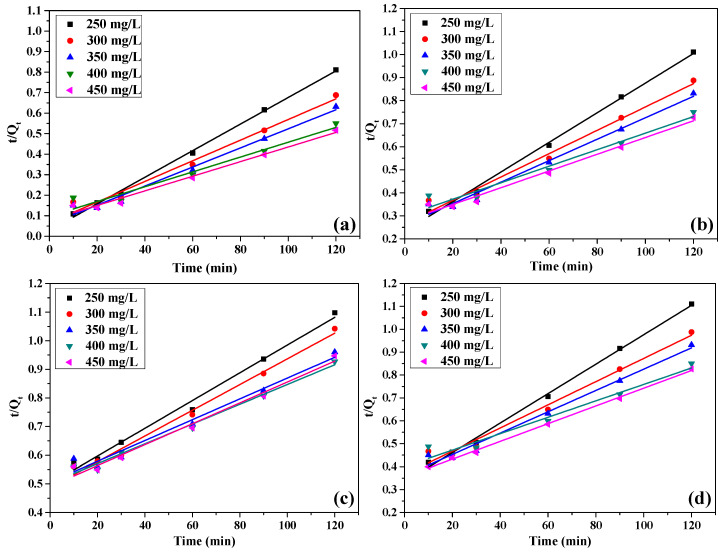
The pseudo-second-order kinetic model for (**a**) GO, (**b**) CFGO-1, (**c**) CFGO-2 and (**d**) CFGO-3 adsorption on cement.

The fitting results of pseudo-first-order and pseudo-second-order kinetic models at different concentrations of GO, CFGO-1, CFGO-2 and CFGO-3 on cement are shown in Figure 7 and Figure 8, respectively. The linear correlation coefficients (R^2^) are exhibited in Table 4. The results showed that R^2^ of pseudo-second-order kinetic model for GO were more satisfactory than pseudo-first-order kinetic model. For CFGOs, although the lengths of the branched-chains were different, all of the R^2^ of the pseudo-second-order kinetic model was more relevant than that of the pseudo-first-order kinetic model.

These results indicate that the GO and CFGO adsorption process cannot be well fitted with the pseudo-first-order kinetic model, but agree with the pseudo-second-order kinetic model. This means that the nature of the adsorption of CFGOs on cement was a chemical-controlling process and the rate-controlling steps [26,28,29], which was the same as GO. For GO, as we reported before, the -COOH on GO reacted with the Ca^2+^ during the hydration process, producing -COO^−^ which could act as the anchor points on the positively charged sites at the surface of cement particles [26,32]. For CFGOs, the adsorption process on the cement also included a chemical reaction. This is attributed to the fact that there remained -COOH on the structure CFGOs, which did not react with -NH_2_ in polyether amine during the chemically functionalized process. Once CFGOs were added into the cement system, the residual -COOH also reacted with the metal cations.

In the case where *C*_0_ was 450 mg/L, as illustrated in Table 5, the pseudo-second-order rate constant of GO was larger than that of CFGOs, and the pseudo-second-order rate constant of CFGOs reduced with the length of branched-chains. This suggested that adsorption capacity of GO on cement was stronger than CFGOs, and the adsorption capacity of CFGOs on cement weakened with the increasing of branched-chain lengths.

### 3.5. Adsorption Isotherms GO/CFGOs on Cement

The equilibrium adsorption state is dynamic in nature, as the amount of adsorbate migrating onto the adsorbent is counterbalanced by the amount of adsorbate migrating back into solution. The relation between the amount adsorbed by an adsorbent and the equilibrium concentration of the adsorbate at a constant temperature in a solid–liquid interface can be expressed by the linearized Langmuir adsorption isotherm and the Freundlich isotherm [31,33,34,35], therefore the adsorption results of GO/CFGOs on cement were fitted with Langmuir and Freundlich isothermal models. The Langmuir isothermal model assumed that the adsorption was a monolayer type on a homogeneous surface [36]. The Langmuir isothermal model can be expressed as:

(4) $$\frac{1}{Q_{e}}=\frac{1}{Q_{em}}+\frac{1}{{bQ}_{em}C_{e}}$$

As another isothermal model, the Freundlich isothermal model was based on the assumption that the adsorbate concentration on the adsorbent surface enhanced with the concentration of adsorbate, and it is usually used to describe the multilayer type and heterogeneous systems [37]. The Freundlich isothermal model can be expressed as:

(5) $${lnQ}_{e}={lnK}_{f}+\frac{1}{n}{lnC}_{e}$$

where *Q_e_* (mg·g^−1^) and *Q_em_* (mg·g^−1^) present the adsorption amount and saturated adsorption amount; *C_e_* (mg·L^−1^) is the equilibrium concentration; *b* represents the constant contingent on the nature of the adsorbate and adsorbent; *K_f_* and *n* are the constants depending upon the adsorption capacity and adsorption amount.

**Figure 9:**
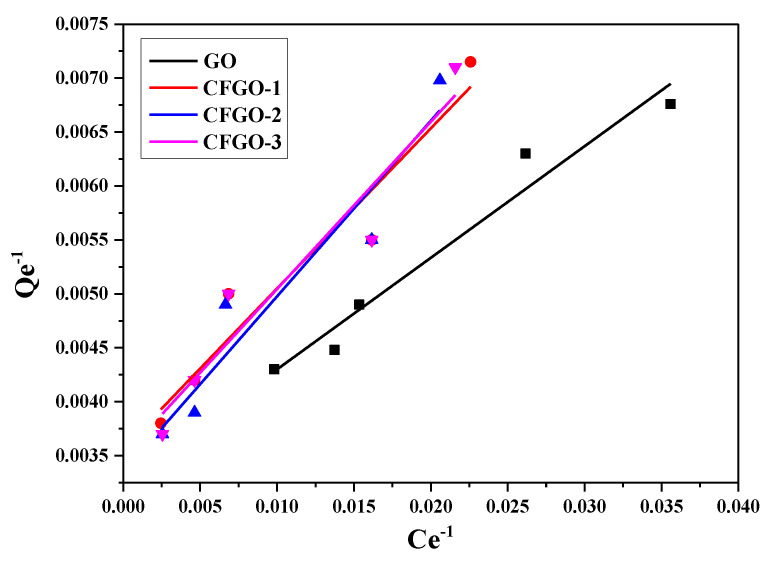
The Langmuir isothermal model for GO and CFGOs adsorption on cement.

**Figure 10:**
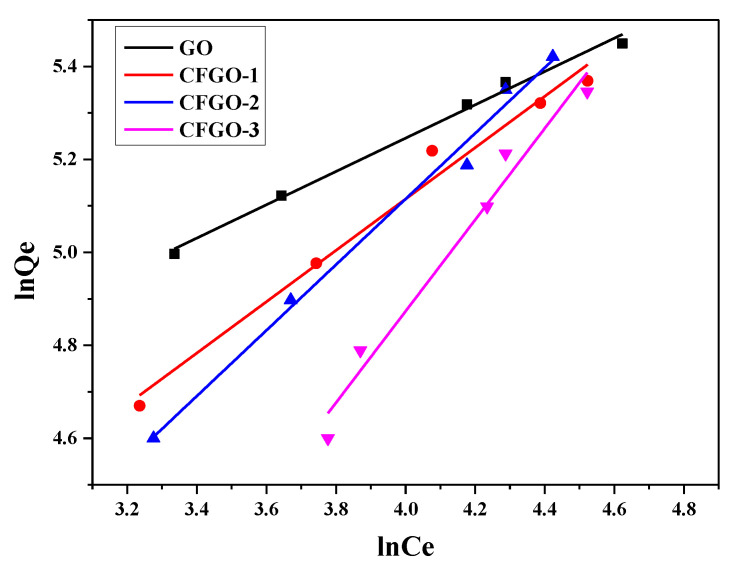
The Freundlich isothermal model for GO and CFGOs adsorption on cement.

The fitting results of Langmuir and Freundlich isothermal models of GO, CFGO-1, CFGO-2 and CFGO-3 adsorbed on cement are respectively plotted in Figure 9 and Figure 10, respectively. The linear correlation coefficients (R^2^) are exhibited in Table 6. The R^2^ of GO for the Freundlich isothermal model was more satisfactory than the Langmuir isothermal model, and R^2^ of CFGOs for the Freundlich model was also closer to 1 than that of the Langmuir model. In other words, the experimental results of GO and CFGOs were fitted better with the Freundlich isothermal model than the Langmuir isothermal model.

The results demonstrate that the adsorption of CFGOs and GO on cement occurred in a heterogeneous manner, and the adsorption of GO and CFGOs on cement could be regarded as a multilayer type [26,29]. As listed in Table 7, it is observed that the values of n and K_f_ of GO adsorbed on cement were larger than that of CFGOs, and these values also reduced with the length of branched-chains. The variation tendency of n and K_f_ values indicated that adsorption capacity of GO on cement was stronger than CFGOs, and the adsorption capacity of CFGOs on cement weakened with the length of branched-chains [38]. These results were consistent with pseudo-second-order rate constant. There were two reasons for the difference in adsorption capacity. Firstly, the chemical reaction between GO and polyether amine consumed -COOH on GO, so that the anchoring effect of CFGOs was weaker than GO. Secondly, the van der Waals force among GO layers on the surface of the cement was strong. However, for CFGOs, the branched-chains provided the steric hindrance effect which weakened the van der Waals force among CFGOs layers, and the steric hindrance effect strengthened with the length of branched-chains.

### 3.6. XPS Spectra of Cement Surface before and after the Adsorption of GO/CFGOs

**Figure 11:**
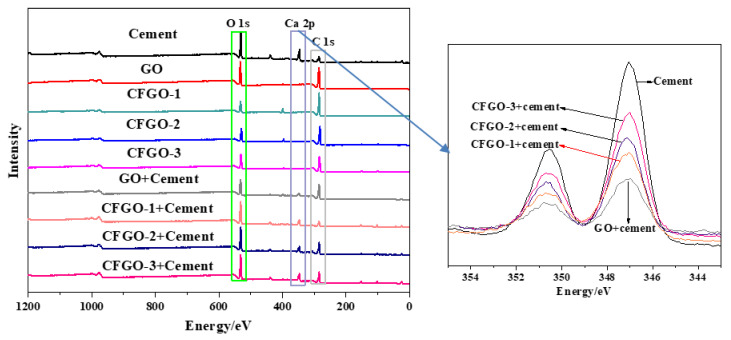
X-ray Photoelectron Spectroscopy (XPS) spectra of cement surface before and after the adsorption of GO/CFGOs.

Figure 11 indicates an XPS survey scan of cement surface before and after the adsorption of GO and CFGOs. GO and CFGOs were mainly constituted of C 1s (signal at 284 eV), O 1s (signal at 532 eV), the peaks of Ca 2p (signal at 347 eV) were observed in pure cement, and the cement after the adsorption of GO and CFGOs. It is interesting to note that the signal intensity of Ca 2p for pure cement was the strongest, and it decreased after GO or CFGO adsorption on the cement. Additionally, the signal intensity of GO adsorbed on cement was weaker than CFGOs. For CFGOs, the signal intensity of CFGO-1 adsorption on cement was weaker than CFGO-2 and CFGO-3, the signal intensity of CFGO-3 was stronger than CFGO-2. These results prove the fact that the adsorption layer thickness of GO adsorbed on cement surface was thicker than CFGOs, and the adsorption layer thickness of CFGOs thinned with the increasing length of branched-chains.

## 4. Illustration of GO and CFGOs with Incorporation of Cement

**Figure 12:**
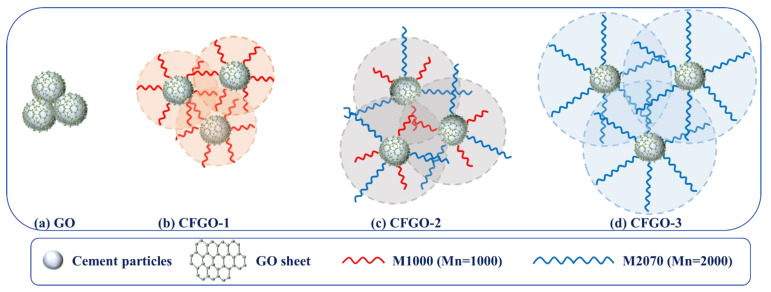
Illustration of GO and CFGOs with incorporation of cement.

The illustration of GO and CFGOs with the incorporation of cement is shown in Figure 12. As displayed in Figure 12a, the aggregation of cement particles was severe, which resulted from the GO adsorbed on cement particles, and the strong van der Waals force among GO layers led to the reduction of the spacing among cement particles. Moreover, Figure 12b–d exhibited the illustration of CFGO-1, CFGO-2 and CFGO-3 with the incorporation of cement. The steric hindrance effect, which was provided by the branched-chains, weakened the van der Waals forces among CFGOs layers. Additionally, the steric hindrance effect strengthened with the increase of branched-chain length. Consequently, the particle spacing of CFGO incorporation with cement was further than that of GO incorporation with cement. These are the essential reasons why CFGOs improved the flowability of cement but GO reduced the flowability of cement.

## 5. Conclusions

Chemically functionalized graphene oxide (CFGO) was obtained through a condensation reaction between graphene oxide (GO) and polyether amine. The main template of CFGOs was the GO sheet, and the branched-chains were polyether amine with different molecular weights. CFGOs improved the flowability, whereas GO reduced the flowability of cement.

The adsorption results of GO and CFGOs were all best fitted with the pseudo-second-order kinetic model and the Freundlich isothermal model. This means that the adsorption of GO and CFGOs on the surface of cement particles both occurred in a heterogeneous manner, and it was multilayer adsorption. Additionally, chemical reactions were engaged in the adsorption process of GO and CFGOs on cement.

The different chemical structures of GO and CFGOs resulted in the distinguished difference in adsorption behavior. For GO, the anchoring effect from -COOH and the strong van der Waals force among the GO layers led to a larger adsorption amount, stronger adsorption capacity and thicker adsorption layer thickness than CFGOs, which resulted in a spacing reduction among cement particles, subsequently leading to the aggregation of cement particles. This is the reason why the introduction of GO reduced the flowability of cement. As for CFGOs, due to the consumption of -COOH by -NH_2_ in polyether amine, the anchoring effect was weaker than GO. The branched-chains provided the steric hindrance effect which weakened the van der Waals force among CFGOs layers, then reduced adsorption amount, weakened adsorption capacity and thinned adsorption layer thickness. Moreover, the steric hindrance effect strengthened with the increase of branched-chain length. The steric hindrance effect endowed the decreased aggregation of cement particles. It is therefore reasonable that chemically functionalized processes that generate CFGOs improved the flowability of cement, and the flowability of cement improved with the increase of branched-chain length.

The branched-chains of CFGOs have significant impact on the flowability and mechanical properties of cement and adsorption behavior on the surface of cement. Other than the length of branched-chains, the performance of cement-based materials also could be adjusted by controlling the category and density of branched-chains. This will be studied in further work.

## Figures and Tables

**Table 1:** Chemical components of cement (%).

Composition	CaO	SiO_2_	Al_2_O_3_	Fe_2_O_3_	SO_3_	MgO	K_2_O	Na_2_O	LOI
Dosage (%)	65.1	21.3	5.1	2.9	1.8	1.1	0.7	0.3	1.7

**Table 2:** The different dosages of the graphene oxide (GO) and polyether amine for CFGOs.

Mark	GO (g)	M1000 (g)	M2070 (g)
CFGO-1	0.068	1.2	0
CFGO-2	0.068	0.6	0.6
CFGO-3	0.068	0	1.2

**Table 3:** Variation of flowability (mm) of cement paste with the different dosages of GO/CFGOs (w/c = 1:3).

	Flowability (mm)/Increase Rate (%)				
Dosage (%)	0.01	0.02	0.03	0.04	0.05
GO	215.1/−2.9	208.4/−5.9	201.3/−9.1	197.1/−11.0	180.3/−18.6
CFGO-1	223.3/0.8	225.1/1.6	231.5/4.5	239.0/7.9	245.2/10.7
CFGO-2	227.6/2.8	231.4/4.5	240.0/8.4	247.6/11.8	253.5/11.4
CFGO-3	237.1/7.0	249.1/12.5	262.5/18.5	270.0/21.9	288.5/30.2

**Table 4:** The correlation coefficients of pseudo-first-order and pseudo-second-order kinetic models for GO/CFGOs adsorption on cement.

	Pseudo–First–Order					Pseudo–Second–Order				
C_0_ (mg/L)	250	300	350	400	450	250	300	350	400	450
GO	0.926	0.883	0.914	0.963	0.976	0.998	0.978	0.980	0.979	0.978
CFGO-1	0.893	0.888	0.745	0.885	0.881	0.996	0.977	0.978	0.955	0.997
CFGO-2	0.741	0.777	0.832	0.846	0.821	0.988	0.988	0.965	0.984	0.979
CFGO-3	0.795	0.875	0.904	0.935	0.950	0.997	0.979	0.982	0.961	0.975

**Table 5:** The pseudo-second-order rate constant of GO/CFGOs adsorption on cement (C_0_ = 450 mg/L).

Rate Constant	GO	CFGO-1	CFGO-2	CFGO-3
K_2_ (g·mg^−1^·min^−1^)	9.86 × 10^−5^	2.38 × 10^−5^	2.11 × 10^−5^	1.36 × 10^−5^

**Table 6:** The coefficient of associations of Langmuir and Freundlich isothermal models for GO/CFGOs adsorption on cement.

Mark	Langmuir	Freundlich
GO	0.950	0.991
CFGO-1	0.892	0.991
CFGO-2	0.894	0.988
CFGO-3	0.901	0.965

**Table 7:** Parameters of Freundlich models for GO/CFGOs adsorption on cement.

Parameters	GO	CFGO-1	CFGO-2	CFGO-3
n	2.79	1.81	1.34	1.01
K_f_	45.24	18.30	9.89	2.57
